# The Mediating Effects of Eating Disorder, Food Addiction, and Insomnia in the Association between Psychological Distress and Being Overweight among Iranian Adolescents

**DOI:** 10.3390/nu12051371

**Published:** 2020-05-11

**Authors:** Chung-Ying Lin, Pauline Cheung, Vida Imani, Mark D. Griffiths, Amir H. Pakpour

**Affiliations:** 1 Department of Rehabilitation Sciences, Faculty of Health and Social Sciences, The Hong Kong Polytechnic University, Hung Hom, Hong Kong; cylin36933@gmail.com (C.-Y.L.); paulinekaka@hotmail.com (P.C.); 2 Pediatric Health Research Center, Tabriz University of Medical Sciences, Tabriz 5166/15731, Iran; dr.v.imani@gmail.com; 3 Nottingham Trent University, International Gaming Research Unit, Psychology Department, Nottingham NG1 4FQ, UK; mark.griffiths@ntu.ac.uk; 4 Social Determinants of Health Research Center, Research Institute for Prevention of Non-Communicable Diseases, Qazvin University of Medical Sciences, Qazvin 3419759811, Iran; 5 Department of Nursing, School of Health and Welfare, Jönköping University, SE-551 11 Jönköping, Sweden

**Keywords:** eating disorder, food addiction, insomnia, psychological distress, excess weight gain

## Abstract

With obesity and excess weight remaining a serious concern worldwide, investigating the mechanisms underlying this is of great importance. Psychological distress is a possible trigger contributing to excess weight for adolescents. Moreover, the association between psychological distress and excess weight may be mediated by eating disorder, food addiction, and insomnia. The present study utilized parallel mediation analysis to assess the aforementioned associations and possible mediation effects among Iranian adolescents. Through stratified and clustered sampling, adolescents (N = 861; mean ± SD age = 15.9 ± 3.2; 372 males) participated and were followed for a one-year period. Excess weight (standardized body mass index, z-BMI); psychological distress (Depression, Anxiety, and Stress Scale-21); eating disorder attitudes (Eating Attitude Test-26); food addiction (Yale Food Addiction Scale for Children); and insomnia (Insomnia Severity Index) were assessed. Eating disorder attitudes, food addiction, and insomnia were significant mediators in the association of psychological distress and z-BMI. Additionally, psychological distress had direct effects on z-BMI. Given that eating disorder attitudes, food addiction, and insomnia showed mediated effects in the temporal association of psychological distress and excess weight, healthcare providers are encouraged to design programs on improving these three mediators to help adolescents overcome excess weight problems.

## 1. Introduction

The issue of childhood obesity and excessive weight gain is of great concern worldwide although plateaued prevalence of childhood overweight has been reported in some countries, especially in the developed countries [1,2]. More specifically, the prevalence of childhood overweight is still high although not necessarily increasing. Recent statistics from the Centers for Disease Control and Prevention (CDC) in the U.S. reported that 13.9% of 2- to 5-year-olds; 18.4% of 6- to 11-year-olds; and 20.6% of 12- to 19-year-olds were overweight or obese [3]. In addition to the Western populations, Asians have similar problems in relation to the high prevalence of being overweight. For example, the prevalence of childhood obesity in Hong Kong is almost 20% for 9- to 12-year-olds [4]. In Iran (where the present study was carried out), the prevalence rate of childhood obesity for 9- to 16-year-olds increased from 5.2% in 2001 to 14.9% in 2007 [5]. With the dramatically increased prevalence of childhood obesity in Iran, studying potential predictors and mediators of excessive weight gain is both important and crucial.

With such relatively high prevalence rates, the health problems caused by obesity and being overweight are of great concern. More specifically, physical, psychological, and social health are all influenced by being overweight or obese. Adverse consequences for physical health include poor fitness and high risks of developing hypertension, cardiovascular disease, and type II diabetes [6]. Negative consequences for psychological health include low self-esteem and negative body image [7,8,9]. Finally, the adverse effects in social health include such consequences as being bullied (e.g., being isolated or teased) [10]. Numerous studies also demonstrate the negative impacts of being overweight on children’s overall health or quality of life (QoL) [10,11,12,13,14,15]. The negative health consequences together with the relatively high prevalence rates of being overweight make the issue of childhood obesity one of the world’s major health concerns. Consequently, healthcare providers globally need to design effective programs to help obese children to lose weight. To aid the design of such programs, potential factors that contribute to childhood overweight should be investigated further.

More specifically, the mechanisms explaining the relationship between psychological distress and excess weight are of importance for both researchers and healthcare providers. With a proposed mechanism, healthcare providers may be able to develop and foster effective intervention based on robust empirical evidence. Therefore, the present authors propose that eating disorder attitudes (or eating disorder behaviors), food addiction, and insomnia could be potential mediators in the association between psychological distress and excessive weight gain. Moreover, Tanofsky-Kraff et al. [16] found that binge eating led to an increase in body fat mass, but they did not test whether binge eating was a mediator between psychological distress and increase in body fat mass. In a later study, Tanofsky-Kraff et al. [17] additionally found that eating disorders in restraint and weight concern domains predicted high body mass index (BMI). However, they did not examine whether eating disorders were mediators in their proposed model. Goldschmidt et al. [18] found that depressive symptoms predicted binge eating but did not examine any mediating effects in their proposed model. Therefore, although it could be hypothesized that eating disorder attitudes (or eating disorder behaviors) are a potential mediator in the association between psychological distress and gaining excess weight, the current literature does not have such empirical evidence.

The studies of “food addiction” in animal models found that the consumption of high-sugar foods leads to behavioral signs of addiction [19,20]. Although “food addiction” has yet to be recognized as a disorder, it was associated with obesity in adults [21]; yet, the impact of food addiction could be substantial on childhood obesity [22]. More specifically, children are more susceptible to being affected by food than adults are due to psychological and neurobiological vulnerabilities during earlier developmental stages [23]. Moreover, the cognition of children is underdeveloped and could be sensitive to developmental transitions [24]. A national study in the USA found that the general public has a strong belief in food addiction as one of the contributing causes to being overweight and subsequent obesity [25]. A recent study found that food addiction was associated with higher BMI in obese children [22]. Consequently, food addiction may be another potential mediator in the temporal association between psychological distress and gaining excess weight. Similar to eating disorder attitudes, the current literature does not have empirical evidence indicating the mediator role of food addiction in the association between psychological distress and gaining excess weight.

Sleep duration and sleep quality are also associated with weight gain. Studies have found that obese individuals can have insomnia and other sleep difficulties [26,27,28]. Some studies have concluded that individuals with less sleep have more opportunity to consume substantial amounts of energy-rich foods (i.e., high proportion of calories from fats or refined carbohydrates) and eat lower amounts of fruits and vegetables than those with more sleep [29,30]. Therefore, sleep problems and/or insomnia may also be a cause of excess weight gain among adolescents. It is tentatively proposed that insomnia could be a mediator in the temporal association between psychological distress and excess weight because psychological distress is a well-known cause for insomnia [31,32]. Nevertheless, similar to eating disorder attitudes and food addiction, current the literature does not have empirical evidence indicating the mediating role of insomnia in the association between psychological distress and gaining excess weight.

**Figure 1:**
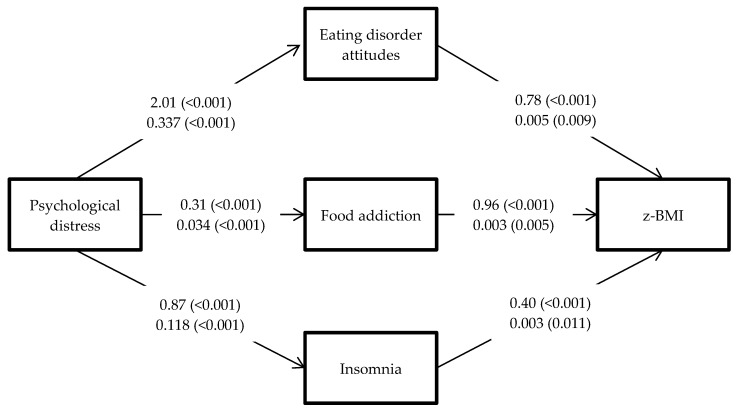
Mediation analysis with the hypothesized mediators of eating disorder attitudes, food addiction, and insomnia. Upper values indicate unstandardized coefficients (*p*-values) from Hayes’ method; lower values indicate unstandardized coefficient (*p*-values) from path analysis. Values from psychological distress to mediators (eating disorder attitudes, food addiction, and insomnia) are direct effects from psychological distress to mediators. Values from mediators to standardized body mass index (z-BMI) are indirect effects of mediators in the relationship of psychological distress and z-BMI.

Given that adolescence is a critical developmental period, where adolescents experience puberty and begin increasing their social interaction with others [33,34], negative health effects at this stage of development might profoundly influence the subsequent life stages of such individuals. Investigating whether modifiable risk factors such as eating disorder attitudes, food addiction, and insomnia are mediators in the relationship between psychological distress and excess weight may help identify important targets for treatment. Although prior studies by the present authors [35,36] have found other psychopathological mechanisms that explain the mediating roles of food addiction, psychological distress, and insomnia for adolescents who are overweight, none of the studies investigated the mechanism explaining the outcome of gaining excess weight. More specifically, Lin et al. [35] investigated on the outcome of quality of life and Ahorsu et al. [36] on binge eating disorder. The outcome of the present study was decreasing, maintaining, or increasing excess weight among Iranian adolescents who were overweight, and the findings will be useful for healthcare providers, especially because such topics are understudied among Iranian adolescents. Consequently, effective programs may be designed to assist overweight adolescents in losing or controlling weight. Consequently, the present study adopted parallel mediation analysis to test the following hypotheses (Figure 1): (i) Eating disorder attitudes will be a significant mediator in the relationship between psychological distress and excess weight; (ii) Food addiction will be a significant mediator in the relationship between psychological distress and excess weight; and (iii) Insomnia will be a significant mediator in the relationship between psychological distress and excess weight.

## 2. Materials and Methods

### 2.1. Participants and Procedure

The research proposal was approved by the Ethics Committee for Biological Research in the Qazvin University of Medical Sciences (IR.QUMS.REC.1398.320). Relevant authorities approved the permissions for sampling. The participants in the present study comprised high school students who were studying in Qazvin, an Iranian city. Forty high schools were randomly selected from 54 high schools in Qazvin. Four classes from each school were randomly selected for participation. Inclusion criteria were (1) age 13–18 years, (2) standardized body mass index (z-BMI) indicates an overweight or above status; that is, over value of 1, and (3) a willingness to participate by providing written informed consent from both the adolescents and at least one of their parents. Adolescents were excluded when informed consent was missing or incomplete. Only adolescents who were overweight or obese were recruited because prior studies have indicated a higher prevalence of food addiction among adolescents who are overweight/obese (5.9%) than among those who are normal weight/underweight (~2.0%) [37].

The adolescents meeting the inclusion criteria were invited to participate in this study which included a one-year follow-up. All participants completed a questionnaire concerning their psychological distress at baseline. One year later, all participants completed other questionnaires concerning eating disorder, food addiction, and insomnia. For both baseline and follow-up assessments, the participants completed paper-based surveys during school time.

A total of 1103 adolescents were initially approached. Of these, 100 adolescents or parents did not provide written informed consent, 39 adolescents were younger than 13 years or older than 18 years, and 103 adolescents did not complete the study measures after giving initial written informed consent. Therefore, the final sample resulted in 861 adolescents. Moreover, no significant differences were found between recruited participants and non-participants in regards to age (15.9 ± 3.2 vs. 15.7 ± 2.19; *p* = 0.40), gender (43.2% males vs. 47.8% males; *p* = 0.14), fathers’ number of years of education (8.6 ± 4.8 vs. 8.1 ± 3.7; *p* = 0.45), and mothers’ number of years of education (6.8 ± 3.9 vs. 6.8 ± 4.0; *p* = 0.69).

### 2.2. Instruments

#### 2.2.1. Measures at Baseline

**Body mass index (BMI) and z-BMI.** In order to calculate the BMI and z-BMI of the participants, their anthropometrics were measured first. All BMIs were measured by school health staff (similar to nurses but a public health specialist) at schools. Moreover, a stadiometer (Seca Model 207, Seca, Hamburg, Germany) was used to measure the heights of all participants to the nearest 0.1 cm. A calibrated digital scale was used to measure their weights to the nearest 0.1 kg. Additionally, the participants did not wear shoes when measuring their heights and weights. Following this, body mass index (BMI) was calculated with Z score of BMI (z-BMI) being determined by using a standardized procedure; that is, z-BMI was calculated according to the World Health Organization’s Child Growth Standards [38]. The height, weight, and BMI of the participants’ parents were also collected using the same methods as for their children.

**Depression, Anxiety, and Stress Scale-21 (DASS-21).** In order to assess psychological distress, the DASS-21 was used. The DASS-21 uses three subscales (each containing seven items) to assess three subtypes of distress, comprising depressive symptoms (sample item: “I felt down-hearted and blue”), anxiety symptoms (sample item: “I was aware of dryness of my mouth”), and stress (sample item: “I found it hard to wind down”). Therefore, the DASS-21 serves as a screening tool to assess self-reported symptoms and is not a clinical diagnostic tool. With the use of a four-point Likert scale scoring from 0 (“did not apply to me at all, never”) to 3 (“applied to me very much, or most of the time, almost always”), the total score of DASS-21 ranges between 0 and 63 by summing all the item responses. A higher level of distress is indicated by a higher DASS-21 total score. Additionally, the Persian DASS-21 has been translated with acceptable linguistic validity with satisfactory internal consistency (α = 0.84–0.91) [39,40] and promising convergent validity [39]. The DASS-21 has been validated utilizing an adolescent sample [41]. Moreover, the Cronbach’s α of the DASS-21 in the present study was 0.89.

#### 2.2.2. Measures at One-Year Follow-Up

**Eating Attitude Test-26 (EAT-26).** In order to assess the risk of eating disorder attitudes (or behaviors), the 26-item EAT-26 was used. The Iranian EAT-26 contains five constructs, including drive for thinness (sample item: “I am terrified about being overweight”), restrained eating (sample item: “I avoid eating when I am hungry”), perceived social pressure to eat (sample item: “I feel that others would prefer if I ate more”), food preoccupation and oral control (sample item: “I feel that food controls my life”), and bulimia (sample item: “I vomit after I have eaten”) [42]. Therefore, the EAT-26 serves as a screening tool to assess self-reported symptoms and is not a clinical diagnostic tool. With the use of a six-point Likert scale scoring from 0 (“never”) to 5 (“always”) converted into a four-point format for calculation (0 = “never, rarely, and sometimes”; 1 = “often”; 2 = “usually”; 3 = “always”), the total score of EAT-26 ranges between 0 and 78 by summing all the item responses. A higher level of disturbance in eating attitudes (i.e., eating disorder) is indicated by a higher EAT-26 score. Additionally, the Persian EAT-26 has been translated and validated with adequate internal consistency (α = 0.61–0.92) [42] and satisfactory convergent validity (r = 0.26 with Beck Anxiety Inventory; 0.42 with Binge Eating Scale) [42]. The EAT-26 has been validated utilizing an adolescent sample [42]. Moreover, the Cronbach’s α of the EAT-26 in the present study was 0.86.

**Yale Food Addiction Scale for Children (YFAS-C).** In order to assess food addiction, the YFAS-C was used. The YFAS-C, a version that is suitable for children and adolescents, is modified from the Yale Food Addiction Scale (YFAS), the adult version for assessing food addiction [43]. The YFAS-C, like the YFAS, was developed using the seven criteria on substance-used disorders listed in the *Diagnostic and Statistical Manual of Mental Disorders, 4th edition, Text revision* (DSM-IV-TR), and a total of 25 items were included in the YFAS-C [44]. The seven criteria and corresponding sample items are (1) activity to obtain, use, and recover (criterion): “When I start eating, I find it hard to stop” (sample item); (2) persistent desire (criterion): “I worry about eating too much food” (sample item); (3) large amount of time spent (criterion): “I eat food all day long” (sample item); (4) given up activities (criterion): “I avoid places where I cannot eat the food I want” (sample item); (5) inability to cut down (criterion): “I eat in the same way even though it is causing problems” (sample item); (6) tolerance (criterion): “I need to eat more to get the good feelings I want” (sample item); and (7) withdrawal (criterion): “When I do not eat certain foods, I feel upset or sick” (sample item). Among the 25 items, 18 are rated using a five-point Likert scale scoring from 0 (“never”) to 5 (“always”) and seven are rated using a dichotomous (“yes/no”) scale. Afterward, all the 25 items apply specific scoring thresholds (i.e., each item has a different threshold) to convert into 0 (“no”) or 1 (“yes”). Two scoring versions (including a symptom count scoring version and a diagnostic scoring version) can be generated using the converted dichotomous scores. More specifically, the symptom count scoring version with a score range between 0 and 7 was used for analyses in the present study. The YFAS-C has satisfactory internal consistency (KR-20 = 0.82) [45]. The Persian YFAS-C was translated by our team using a standard method in translation (including forward and back translations, reconciliation, panel reviewing, cultural adaptation, and pilot testing) to ensure its linguistic validity. Moreover, the KR-20 of the YFAS-C in the present study was 0.80.

**Insomnia Severity Index (ISI).** In order to assess the severity and effects of insomnia, the seven-item ISI was used. The ISI only had one construct of insomnia (sample item: How satisfied/dissatisfied are you with your current sleep pattern?). With the use of a five-point Likert scale scoring from 0 (“no problem”) to 4 (“very severe problem”), the total score of ISI ranges between 0 and 28 by summing all the item responses. Different levels of insomnia can therefore be determined using the total score, where 0–7 indicates absence of insomnia, 8–14 indicates sub-threshold insomnia, 15–21 indicates moderate insomnia, and 22–28 indicates severe insomnia [46]. Consequently, severe insomnia is indicted by a higher ISI score. Additionally, the Persian ISI was translated and validated with acceptable internal consistency (α = 0.82 and 0.87), promising test–retest reliability (intraclass correlation coefficient of 0.84 in a two-week interval), and satisfactory convergent validity [47]. The ISI has been validated utilizing an adolescent sample [48]. Moreover, the Cronbach’s α of the ISI in the present study was 0.83.

### 2.3. Data Analysis

Before conducting the formal analyses, skewness and kurtosis were used to understand the data’s distribution, and Mardia’s test was used to assess the multivariate normality of the present data. The results showed that univariate skewness (−0.25–1.21) and kurtosis (−0.81–4.43) were within acceptable ranges. Mardia’s test was not significant (*p* = 1.23), suggesting the presence of normality at the multivariate level. Therefore, no transformations were used to address the distribution issue. Moreover, Little’s test indicated that the missing data in the follow-up assessments completed were missing at random (χ^2^ = 71.035, *df* = 145, *p* = 1.00) and the full information maximum-likelihood method was used to impute data. The correlations between the studied variables (z-BMI, psychological distress, eating disorder, food addiction, and insomnia) were first examined using Pearson’s correlations. Then, the mediation model was constructed as follows: z-BMI was the dependent variable; psychological distress was the independent variable; eating disorder attitudes, food addiction, and insomnia were the mediators; age, gender, fathers’ education, parents’ BMI, and baseline z-BMI were the controlled variables. Age, gender, parents’ BMI, and baseline z-BMI were controlled because prior studies have defined them as potential confounders in weight status [49]; fathers’ education was controlled because it is a relevant confounder under the Iranian context [35,50]. More specifically, the z-BMI rather than the BMI was used in the data analysis because z-BMI can provide clearer information as to whether clinically significant changes can be observed. By using Cohen’s effect size, a change of 0.2 in the z-BMI indicates a clinically significant change [11,15,51]. Moreover, the mediation model was assessed using Hayes’ Model 4 in the PROCESS macro for SPSS and a bootstrapping method with 10,000 resamples was used [52].

In addition to the Hayes’ Model 4, a path analysis incorporated with structural equation modeling (SEM) was performed to further understand the mediational relationships because such practice allows theory development [53]. In the path analysis, a maximum-likelihood estimator was used for parameter estimations and several fit indices were adopted to examine the data-model fit. The fit indices indicating good fit include comparative fit index (CFI) >0.9, Tucker–Lewis index (TLI) >0.9, root mean square error of approximation (RMSEA) <0.08, and standardized root mean square residual (SRMR) <0.08 [54]. Moreover, bias-corrected bootstrapping with 5000 iterations [55] was used to test mediational relationships. Path analysis incorporated with SEM was conducted using AMOS version 24.0 (IBM Corporation, Armonk, NY, USA).

Although the best method to test the mediation model is to assess mediators and final outcomes in different time points, this present study simultaneously assessed mediators and final outcomes. Therefore, the mediation model was not optimal. However, findings from current simultaneously assessed mediators and final outcomes can provide evidence for future studies to further study their causal relationship. Indeed, Collins and Maxwell [56] have claimed that using two waves of data (or a “half-longitudinal design” as termed by Collins and Maxwell) still provides partial information in the mediation analysis. More specifically, when the independent variable is measured at baseline and mediator and outcome variable are concurrently measured at follow-up, the estimation from outcome variable to mediator is robust. The problem of such a design (i.e., a concurrently measured mediator and outcome variable) is the biased estimate in the effect of the mediator on the outcome variable. However, such a design can be used if the resources are limited. Therefore, the present authors adopted the method proposed by Collins and Maxwell that assessed mediators and final outcomes simultaneously.

## 3. Results

Among the 861 adolescents aged between 13 and 18 years, less than half were males (n = 372; 43.2%). In terms of their z-BMI, it was 2.4 ± 0.6 at baseline and 2.4 ± 0.7 one year later. Moreover, over 60% of the participants changed z-BMI with an absolute difference of 0.2 or higher. Therefore, z-BMI change among sample participants had sufficient variability to test the hypothesized relationships. Table 1 also reports the participants’ scores in psychological distress, eating disorder, food addiction, and insomnia. Among the 861 adolescents, 63 did not complete the follow-up assessment (an attrition rate of 7.3%).

All the bivariate correlations between z-BMI (including baseline and follow-up assessments), psychological distress, eating disorder attitudes, food addiction, and insomnia were significant (*p* < 0.01) and ranged between 0.13 and 0.47 (Table 2). The mediation analysis with Hayes’ method (Table 3; Figure 1) verified that psychological distress had a direct effect on eating disorder attitudes (unstandardized coefficient [B] = 2.01; *p <* 0.001) and eating disorder attitudes had an indirect effect on z-BMI (B = 078; 95% confidence interval (CI) = 0.41, 1.20); psychological distress had a direct effect on food addiction (B = 0.31; *p* < 0.001) and food addiction had an indirect effect on z-BMI (B = 0.96; 95% = 0.54, 1.43); psychological distress had a direct effect on insomnia (B = 0.87; *p* < 0.001) and insomnia had an indirect effect on z-BMI (B = 0.40; 95% CI = 0.10, 0.78). Similar direct and indirect effects were found in the path analysis (Table 4; Figure 1). Moreover, the path analysis had satisfactory fit indices (χ^2^ = 2.38; *df* = 1; CFI = 1.00; TLI = 0.99; RMSEA = 0.040; SRMR = 0.003). Therefore, the mediational relationships proposed in the present study were all supported. 

## 4. Discussion

To the best of the present authors’ knowledge, this is the first study to use parallel mediation analysis to assess whether the temporal association between psychological distress and excess weight is mediated via different mediators (i.e., eating disorder attitudes, food addiction, and insomnia). The study hypotheses were supported by parallel mediation analyses in both Hayes’ method and path analysis method. The results verify that psychological distress was associated with the excess weight measured using z-BMI one year later, and that all three mediators were all significant. Additionally, the highest level of the mediated effects was food addiction (B = 0.96), followed by eating disorder attitudes (B = 0.78) and insomnia (B = 0.40).

The three mediators help explain why psychological distress could be one of the contributors in excess weight. Aligned with prior research, the results of the present study demonstrated that higher psychological distress is related to higher risk of eating disorder attitudes [57]. Eating disorder attitudes have been found to contribute to increased body fat mass [16]. Therefore, the adolescents with high level of psychological distress may gain excess weight because of the high risk of eating disorder attitudes. Another mediator related to eating—food addiction—was also a significant mediator in the findings presented here. It is unsurprising that individuals with food addiction are overweight or obese [25]. Because individuals tend to consume high-energy food when they are under pressure [58], some of them may develop a food addiction and subsequently gain weight. Therefore, food addiction appears to be another significant mediator in the association of psychological distress and excess weight.

In terms of insomnia, some studies have found that insomnia is associated with excess weight [59,60], and prior research has proposed that psychological distress is a cause of insomnia [31]; i.e., an individual with psychiatric or mood problems has greater difficulties in sleeping compared with an individual without such problems. Unfortunately, the effects of insomnia may be exacerbated by psychological problems [61,62] and can lead to excess weight via unhealthy eating when the individual cannot get sleep, and where they possibly consume energy-rich foods [29].

### Limitations

The present study has several limitations. First, the present study’s findings might not be generalized to other countries, especially western countries, given that the study sample was recruited in Iran only. Because different countries have different cultures in terms of sleep and eating, future studies are warranted to investigate whether the mediation model supported in the present study can be extended to other countries with different styles of sleep and eating. Second, some important confounders to the mediators (for example, sleep hygiene behaviors for insomnia) were not collected and controlled in the proposed mediation model. Therefore, interpretations of the results should be treated with some caution given the possible effects from such uncontrolled confounders. Nevertheless, the findings of the present study can provide some baseline data and/or markers for future studies to control for confounding variables. Third, most of the data collected in the study were self-reported in nature. Healthcare providers and researcher should be cautioned by the well-known biases such as social desirability bias or memory recall bias. Future studies using more objective instruments (e.g., actigraphy to assess insomnia) are therefore needed to corroborate the findings presented here. Fourth, the present study only recruited adolescents with a weight issue because of high food addiction prevalence. However, the tested mediational analyses may also fit in those without overweight/obesity. Therefore, future studies may want to corroborate the present study’s findings on a sample with diverse weight conditions. Finally, and importantly, the mediators (eating disorder, food addiction, and insomnia) and the final outcome (z-BMI) were assessed simultaneously (i.e., one year after baseline). Therefore, it is unclear whether the mediators truly mediated the association between psychological distress and z-BMI, or the mediators (e.g., sedentary behavior) were simply confounded with z-BMI. Future studies are therefore needed to further investigate this relationship.

## 5. Conclusions

In conclusion, the present study found three important mediators (eating disorder attitudes, food addiction, and insomnia) in the temporal association between psychological distress and excess weight. Healthcare providers may gain insight from such findings and help foster appropriate and effective programs to prevent excess weight problems among adolescents. For example, underlying problems (psychological distress) or the mediated problems (eating disorder attitudes, food addiction, and insomnia) may be tackled by the adolescents through assistance from healthcare providers. Consequently, the adolescents may prevent excessive weight gain. However, the present findings should be interpreted with caution. More specifically, mediators and follow-up weight status were collected simultaneously. Therefore, the causal relationships between mediators (eating disorder attitudes, food addiction, and insomnia) and excess weight are unclear. Consequently, future studies are needed to examine the postulation that programs for improving mediated problems can address weight problems among adolescents.

## Figures and Tables

**Table 1:** Characteristics of the study participants (N = 861).

	Mean ± SD or n (%)
Age (Year)	15.9 ± 3.2
Gender (Male)	372 (43.2)
Number of years education (father)	8.6 ± 4.8
Number of years education (mother)	6.8 ± 3.9
z-BMI at baseline	2.4 ± 0.6
z-BMI at one year follow-up	2.4 ± 0.7
Mothers’ BMI (kg/m^2^)	40.2 ± 5.6
Fathers’ BMI (kg/m^2^)	37.8 ± 5.7
Psychological distress ^a^	31.1 ± 15.9
Eating Attitude Test-26	23.2 ± 10.4
Food addiction ^b^	2.8 ± 1.3
Insomnia Severity Index	9.4 ± 4.3

^a^ Assessed using the Depression, Anxiety, and Stress Scale-21. ^b^ Assessed using symptom counts on the Yale Food Addiction Scale for Children.

**Table 2:** Pearson′s correlation matrix of the variables of interest.

	Time 2 z-BMI	Time 1 z-BMI	Psychological Distress ^1^	Eating Disorder Attitudes ^2^	Food Addiction ^3^	Insomnia ^4^	Mothers’ BMI	Fathers’ BMI	Age	Gender	Fathers’ Education
z-BMI at time 2	1	0.466 **	0.278 **	0.201 **	0.305 **	0.300 **	0.111 *	0.129 *	0.095 *	0.045	0.136 **
z-BMI at time 1		1	0.126 **	0.186 **	0.152 **	0.197 **	0.105 **	0.087	0.073	0.035	
Psychological distress ^1^			1	0.395 **	0.410 **	0.348 **	0.060	0.66	0.015	0.022	0.031
Eating disorder attitudes ^2^				1	0.295 **	0.219 **	0.093 *	0.089 *	0.033	0.005	0.049
Food addiction ^3^					1	0.303 **	0.081 *	0.070	0.247 **	0.171 **	0.039
Insomnia ^4^						1	0.010	0.015	0.090 **	0.159 **	0.085 *
Mothers’ BMI							1	0.96 **	0.038	0.032	0.125 **
Fathers’ BMI								1	0.036	0.034	0.134 **
Age									1	0.014	0.021
Gender										1	−0.042
Fathers’ education											1

^1^ Assessed using the Depression, Anxiety, and Stress Scale-21. ^2^ Assessed using the Eating Attitude Test-26. ^3^ Assessed using the symptom counts on Yale Food Addiction Scale for Children. ^4^ Assessed using the Insomnia Severity Index. * *p*-value < 0.05; ** *p*-value < 0.01.

**Table 3:** Models of the effect of adolescents′ psychological distress on body mass index (BMI) with mediators of insomnia, food addiction, and eating disorder attitudes.

	Unstand. Coeff.	SE or (Bootstrapping SE)	t-Value (*p*-Value)	Bootstrapping LLCI, ULCI
Total effect of psychological distress on z-BMI	3.40	0.45	7.62 (<0.001)	
Direct effect of psychological distress on z-BMI	1.26	0.42	2.67 (0.003)	
Direct effect of psychological distress on mediators				
Eating disorder attitudes	2.01	0.37	5.44 (<0.001)	
Food addiction	0.31	0.04	8.82 (<0.001)	
Insomnia	0.87	0.14	5.87 (<0.001)	
Indirect effect of psychological distress on z-BMI				
Total indirect effect	2.14	(0.37)	5.78 (<0.001)	1.43, 2.87
Through eating disorder attitudes	0.78	(0.20)	3.90 (<0.001)	0.41, 1.20
Through food addiction	0.96	(0.23)	4.17 (<0.001)	0.54, 1.43
Through insomnia	0.40	(0.17)		0.10, 0.78

Note: Age, gender, father’s education, parents’ BMI, and baseline z-BMI were adjusted for the model. Psychological distress was assessed using the Depression, Anxiety, and Stress Scale-21; eating disorder attitudes were assessed using the Eating Attitude Test-26; food addiction was assessed using the Yale Food Addiction Scale for Children; insomnia was assessed using the Insomnia Severity Index. Unstand. Coeff. = unstandardized coefficient. SE = standard error. LLCI = lower limit in 95% confidence interval. ULCI = upper limit in 95% confidence interval.

**Table 4:** Direct, indirect, and total effects of the path analysis.

Path	B (Bootstrapping SE)	β	LL	UL
**Direct effects**				
Psychological distress → z-BMI	0.005 (0.001)	0.054 **	0.001	0.009
Psychological distress → food addiction	0.034 (0.003)	0.405 ***	0.028	0.040
Psychological distress → eating disorder attitudes	0.337 (0.043)	0.400 ***	0.275	0.420
Psychological distress →insomnia	0.118 (0.021)	0.331 ***	0.079	0.149
**Indirect effects**				
Psychological distress → food addiction →z-BMI	0.003 (0.001)	0.036 **	0.002	0.005
Psychological distress → eating disorder attitudes →z-BMI	0.005 (0.001)	0.058 **	0.003	0.007
Psychological distress → insomnia z-BMI	0.003 (0.001)	0.032 *	0.001	0.004
Psychological distress → food addiction, eating disorder attitudes, and insomnia→z-BMI	0.011 (0.002)	0.041 **	0.008	0.014
**Total effects**				
Psychological distress →z-BMI	0.015 (0.003)	0.172 *	0.010	0.019

Note. Age, gender, father education and baseline z-BMI were controlled in the hypothesized model. B = unstandardized path coefficient; SE = standard error; β = standardized path coefficient; LL = lower limit at 95% confidence interval of path coefficient; UL = upper limit at 95% confidence interval of path coefficient. * *p* < 0.05 ** *p* < 0.01 *** *p* < 0.001.
